# Intestinal Lymphangiectasia: A Rare Etiology of Gastrointestinal Bleeding

**DOI:** 10.1002/ccr3.70637

**Published:** 2025-08-07

**Authors:** Mohamed A. Baghi, Abdulwahab Hamid, Ahmed Mohamed Badi, Muneera Jassim Al‐Mohannadi

**Affiliations:** ^1^ General Internal Medicine Hamad Medical Corporation Doha Qatar; ^2^ Qatar University College of Medicine (QU) Doha Qatar; ^3^ Weill Cornell Medicine‐Clinical Medicine, (WCM‐CM) Doha Qatar; ^4^ Al‐Khor Hospital Al Dhakira Qatar

**Keywords:** gastrointestinal bleeding, intestinal lymphangiectasia, jejunal lesion, malabsorption

## Abstract

Intestinal lymphangiectasia (IL) is a rare condition characterized by the blockage of lymphatic fluid draining from the small intestine. It commonly presents with symptoms such as malabsorption, diarrhea, and hypoproteinemia. Bleeding from IL is an infrequent manifestation. We report a rare case of bleeding intestinal lymphangiectasia in a patient who presented with symptomatic anemia and melena. Enteroscopy identified a focal mucosal lesion without active bleeding, while video capsule endoscopy revealed a mucosal lesion with active blood oozing in the proximal jejunum. Subsequent biopsy results confirmed focal lymphangiectasia with vascular congestion. The patient underwent laparoscopic resection of the jejunal lesion. Follow‐up evaluations at 3 and 6 months showed no recurrence of symptoms.

**Summary**

- This case highlights a rare presentation of intestinal lymphangiectasia (IL) manifesting as gastrointestinal bleeding.
- Video capsule endoscopy was crucial in localizing the lesion; histopathological examination is important to confirm the diagnosis, and surgical resection may provide a definitive cure.
- IL should be included in the differential diagnosis when investigating unexplained gastrointestinal bleeding.

## 1 Introduction

Intestinal lymphangiectasia can be observed throughout the gastrointestinal (GI) tract, often remaining asymptomatic [1]. In contrast, pathologic lymphangiectasia leads to a range of GI symptoms, including abdominal pain, steatorrhea, ascites, and in rare cases, gastrointestinal bleeding [2, 3]. Diffuse lymphangiectasia may result from conditions such as intestinal tuberculosis or parasitic infections, both of which disrupt lymphatic flow and contribute to protein‐losing enteropathy [4]. In rare instances, intestinal lymphangiectasia (IL) can present with either overt or occult gastrointestinal bleeding [4, 5]. Endoscopic findings typically include whitish spots, specks, or yellowish, well‐defined, raised mucosal or submucosal lesions [5, 6]. Only a limited number of cases of small bowel bleeding associated with IL have been reported [7, 8]. Here, we present a case of jejunal lymphangiectasia diagnosed using video capsule endoscopy and enteroscopy.

## 2 Case History/Examination

A 51‐year‐old male with a medical history of diabetes mellitus (DM), coronary artery disease (CAD), and chronic kidney disease (CKD) was referred for gastroscopy and colonoscopy as part of the evaluation for anemia. The patient had been suffering from anemia since 2015 but had no history of overt gastrointestinal bleeding. He underwent gastroscopy and colonoscopy, which showed unremarkable results. Further investigations, including hemoglobin electrophoresis, were unremarkable except for iron deficiency. The patient was treated with iron supplements for 3 months, after which his hemoglobin (Hb) level improved to 13 g/dL. One year later, the patient re‐presented with a history of generalized fatigability and shortness of breath on minimal exertion for a duration of 1 month. Physical examination was notable for findings of anemia, including marked pallor with pale mucous membranes and conjunctiva. Other system examinations were unremarkable.

## 3 Investigations and Treatment

Laboratory investigations show a significant drop in hemoglobin to 6.1 g/dL, with a hematocrit of 23.6 (40%–50%), mean corpuscular volume (MCV) of 71 (83–101 fL) a white blood cell count of 11,300/μL (4–10 uL), and a platelet count of 209,000/μL (150–400 μL). Prothrombin time is 13 s (INR 1.1) activated partial thromboplastin time (aPTT) 28.8 s (25–36 Seconds), protein level is 80 g/dL (66–87 g/dL), albumin is 40 g/dL (35–50 g/L), and stool ova and parasites are negative. Repeat gastroscopy was unremarkable, while colonoscopy revealed altered blood in the right colon and terminal ileum, without any mucosal lesions.

**FIGURE 1:**
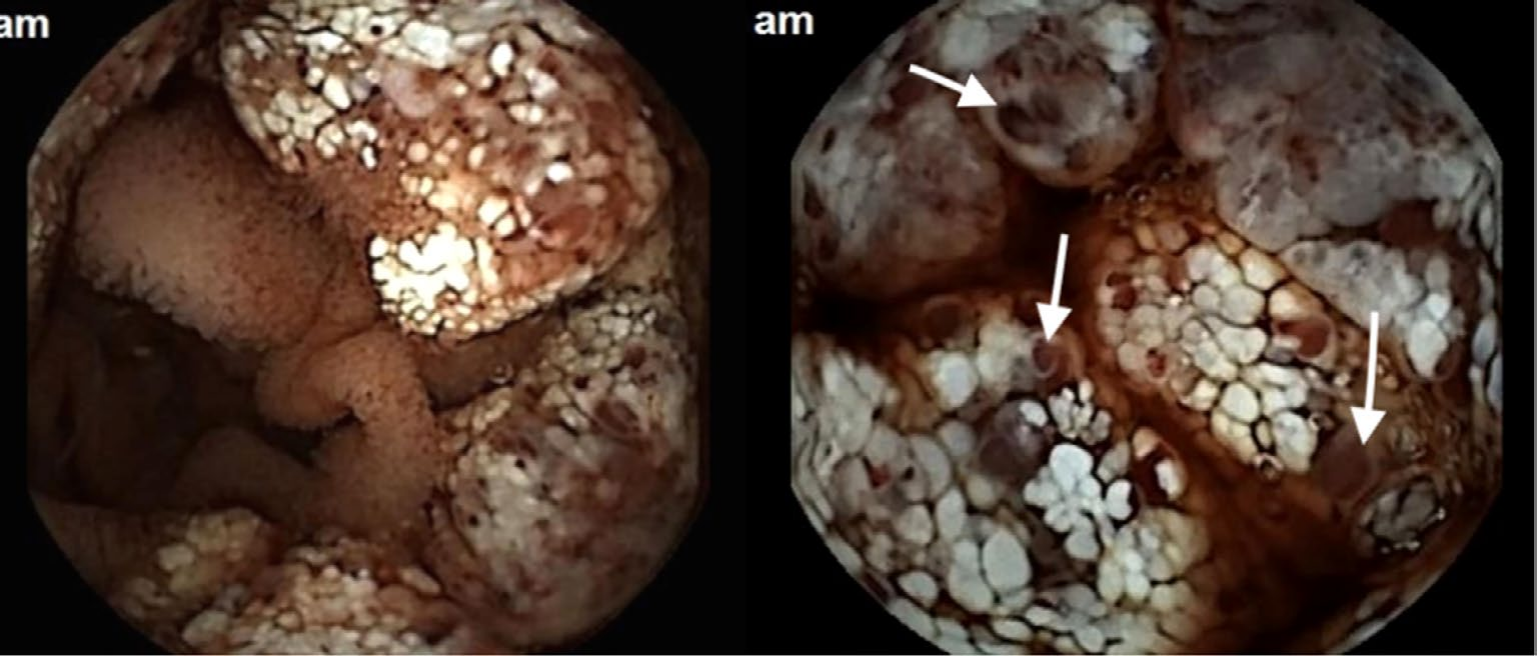
Capsule endoscopy reveals intestinal lymphangiectasia, with jejunal lesions displaying white, nodular villi and active blood oozing (White arrows).

**FIGURE 2:**
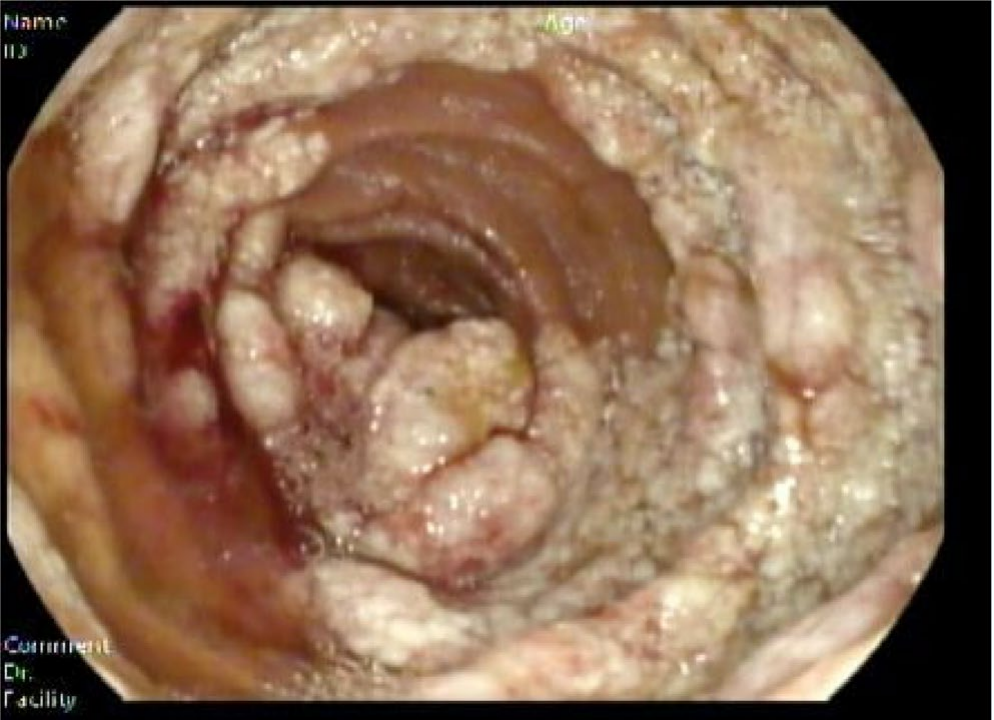
Enteroscopy shows polypoid mucosa covered with enlarged whitish villi.

A CT enterography revealed no significant abnormalities. Subsequently, a video capsule endoscopy (IntroMedic, Seoul, South Korea) was performed, which identified a mucosal lesion in the proximal jejunum with active bleeding (Figure 1). This was followed by enteroscopy, which confirmed a focal mucosal lesion with no active bleeding (Figure 2). Biopsy samples were obtained for histopathological evaluation, and the patient was given a blood transfusion before being discharged to await the results.

**FIGURE 3:**
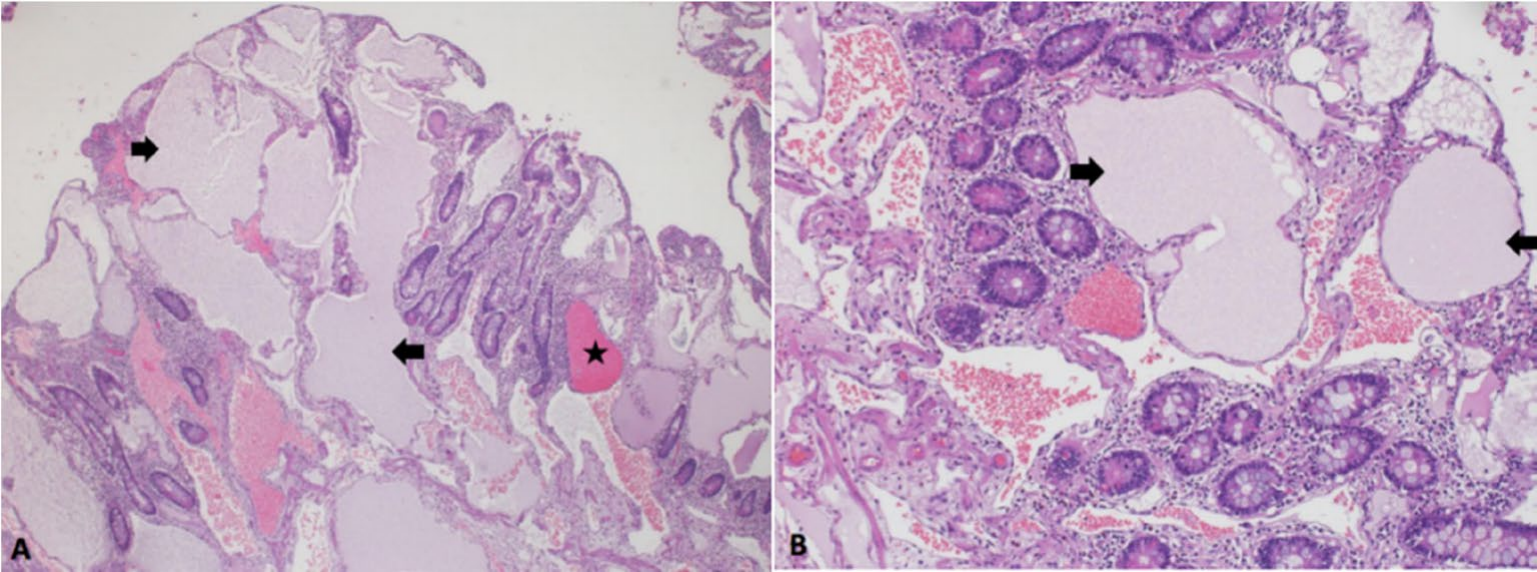
(A, B) Histology of mucosal tissue in the jejunum shows blunting of normal villi by severely dilated mucosal and submucosal lymphatic vessels (Black arrows) with vascular congestion (The Star). (A) HE, × 100. (B) HE, × 200.

A week later, the patient returned to the Emergency Department (ED) with melena and a further drop in his Hb level. After counseling, he underwent laparoscopic resection of the jejunal lesion, which was performed without complications. The histopathological examination revealed focal lymphangiectasia with vascular congestion (Figure 3A,B).

## 4 Outcome and Follow‐Up

The patient was followed up at 3 and 6 months, during which he remained asymptomatic, and his Hb level had stabilized at 13.5 g/dL with a normal iron profile.

## 5 Discussion

Intestinal lymphangiectasia (IL) is a rare disorder within the group of protein‐losing enteropathies, characterized by abnormal dilation of lymphatic vessels in the gastrointestinal tract [1]. IL most frequently affects the duodenum, though it can occur anywhere along the gastrointestinal tract [1, 2]. The condition may be congenital or acquired and is frequently associated with various systemic conditions, including primary lymphatic disorders and gastrointestinal infections [1, 2, 9]. The majority of cases are asymptomatic or present with non‐specific gastrointestinal symptoms including but not limited to generalized fatigability, abdominal pain, weight loss, peripheral edema, diarrhea, and pleural effusion [9]. This case highlights an atypical presentation of IL, where gastrointestinal bleeding was the primary manifestation, without any signs of lower limb swelling, diarrhea, or ascites. Several mechanisms have been proposed to explain the pathophysiology of bleeding lymphangiectasias. Obstruction of normal chyle flow from the small intestine can increase intraluminal pressure enough to trigger opens dormant lymphatic‐venous connections; as a result, the pressure gradient may also open latent lymphatic‐arterial connections. These openings into a higher‐pressure closed system can cause retrograde blood flow into the lymphatics [3, 4, 5]. This will lead to rupture of blood‐filled, dilated lymphatics, resulting in intestinal bleeding, as was evident in our patient, who initially presented with anemia and eventually developed melena and a significant drop in hemoglobin. To the best of our knowledge, few cases of bleeding jejunal lymphangiectasia have been reported [6, 7]. In contrast, our patient exhibited a more diffuse and circumferential lesion compared to the previous cases. Diagnosis of IL can be challenging due to its subtle presentation and the nonspecific nature of symptoms [7]. The traditional diagnostic modalities, such as gastroscopy and colonoscopy, may appear inconclusive, as was observed in our case. These procedures often fail to identify the underlying cause of the anemia or bleeding. In our patient, initial endoscopic evaluations did not provide any clue to explain the anemia, and only after the use of advanced imaging techniques, including video capsule endoscopy (VCE) the jejunal lesion identified. Notably, our study highlighted a case in which the patient also had chronic kidney disease (CKD) [5]. However, our literature review did not uncover any prior studies indicating a direct association between CKD and an increased risk of bleeding in cases of lymphangiectasia. This finding raises interesting questions about possible connections between CKD and the clinical manifestation of lymphangiectasia.

The management of IL depends on the severity of the clinical presentation. In many cases, IL is asymptomatic and may be managed conservatively [2]. Endoscopic interventions or surgical resection are implemented in cases with significant or recurrent bleeding [2, 3, 9]. In our patient, despite initial stabilization with iron supplementation and blood transfusions, he ultimately required laparoscopic resection of the jejunal lesion. The patient showed significant clinical improvement after the surgery and was asymptomatic at 3‐ and 6‐month follow‐up, with his hemoglobin levels returning to normal.

## 6 Conclusion

Our case highlights an unusual presentation of intestinal lymphangiectasia, where gastrointestinal bleeding was the primary symptom. IL should be considered in the differential diagnosis of unexplained gastrointestinal bleeding, especially when other causes have been excluded. Capsule endoscopy is useful to localize the disease, and surgical resection may provide a definitive cure.

## Author Contributions

**Mohamed A. Baghi:** writing – review and editing. **Abdulwahab Hamid:** writing – original draft, writing – review and editing. **Ahmed Mohamed Badi:** writing – original draft, writing – review and editing. **Muneera Jassim Al‐Mohannadi:** investigation, writing – original draft, writing – review and editing.

## Consent

This case was approved by Hamad Medical Corporation's Medical Research Center MRC‐04‐24‐349, and patient consent has been signed and collected in accordance with the journal's patient consent policy.

## Conflicts of Interest

The authors declare no conflicts of interest.

## Data Availability

Data openly available in a public repository that issues datasets with DOIs.
